# Endoscopic negative-pressure therapy for anastomotic leaks after upper gastrointestinal surgery: systematic review and meta-analysis

**DOI:** 10.1007/s00464-026-12790-w

**Published:** 2026-04-20

**Authors:** Santiago Alonso Gómez, María Donat Garrido, Carlos Miliani Molina, Sagrario Martínez Cortijo, Elías Rodríguez Cuellar, Eduardo Ferrero Herrero, Javier Dagnesses-Fonseca

**Affiliations:** 1 Esophagogastric Section, Department of General and Digestive Surgery, Fundacion Alcorcon Hospital, Madrid, Spain; 2 Honorary Tutor, Department of Surgery, Rey Juan Carlos University, Madrid, Spain; 3 Department of General and Digestive Surgery, Hospital Universitario de Mostoles, Madrid, Spain; 4 Esophagogastric Section, Department of General and Digestive Surgery, Hospital Universitario de Fuenlabrada, Madrid, Spain; 5 Esophageal Diseases Unit, Hospital Quirónsalud Sur, Alcorcón, Spain; 6 Esophagogastric Unit, Department of General and Digestive Surgery, Hospital Universitario 12 Octubre, Madrid, Spain; 7 Department of General and Digestive Surgery, Clínica Rotger (Quirónsalud), Illes Balears Palma, Spain

**Keywords:** Anastomotic leak, Endoscopic vacuum therapy, Esophagogastric surgery, Bariatric surgery, Meta-analysis, Negative-pressure therapy

## Abstract

**Background:**

UGI anastomotic leaks are severe postoperative complications associated with substantial morbidity, mortality, and prolonged hospitalization. EVT is a minimally invasive alternative to conventional approaches, but evidence has not been comprehensively synthesized.

**Methods:**

This systematic review and meta-analysis was reported in accordance with the PRISMA 2020 statement. Observational studies (January 2015–January 2025) identified in PubMed, Embase, Cochrane, and LILACS. Adults with UGI anastomotic leaks treated with EVT were eligible. Random-effects models pooled proportions using the Freeman–Tukey double-arcsine transformation; sensitivity analyses used a logit transformation. Pre-specified subgroup analyses stratified by surgical indication (oncologic, bariatric, mixed), leak location (intrathoracic vs intra-abdominal), and risk of bias. Risk of bias was appraised using the Joanna Briggs Institute (JBI) critical appraisal checklist for case series and certainty of evidence with GRADE.

**Results:**

Thirty-one studies (*n *= 767) were included. The pooled clinical success was 87% (95% CI, 82–91%) and remained high in sensitivity analyses (84–89%). No material differences were observed across oncologic, bariatric, or mixed series. Intrathoracic leaks showed lower success than intra-abdominal leaks (79 vs 95%). Thirty-day mortality was 7%, primarily in complex clinical contexts. Heterogeneity was substantial, and small-study effects were suspected. Overall certainty of evidence was rated low owing to observational design and risk of bias.

**Conclusions:**

Across observational studies, EVT achieved high clinical success for UGI anastomotic leaks with low short-term mortality. Prospective cohorts and randomized trials are needed to refine indications, identify predictors of response, and assess cost-effectiveness.

**Graphical abstract:**

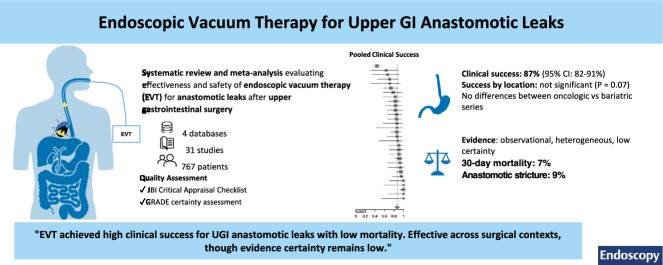

**Supplementary Information:**

The online version contains supplementary material available at 10.1007/s00464-026-12790-w.

Anastomotic leaks are serious complications after upper gastrointestinal (UGI) surgery and are associated with substantial morbidity, mortality, and healthcare costs [1, 2]. In malignancies of the UGI tract, leaks may adversely affect oncologic outcomes, including recurrence and survival [3]. Endoscopic vacuum therapy (EVT) has emerged as a minimally invasive therapeutic option; despite favorable case series and low reported rates of adverse events, adoption remains uneven across centers [4].

EVT applies the principles of negative-pressure wound therapy to intraluminal defects: continuous suction is used to promote granulation, evacuate infected secretions, reduce edema, and improve local perfusion [5]. Available systems generally involve placement of a sponge connected to continuous negative pressure within or adjacent to the defect to facilitate tissue repair and seal the leak [6]. Observational evidence suggests that EVT is effective for anastomotic leaks and may be associated with lower mortality and complication rates than alternatives such as stent therapy; however, because most data are retrospective, estimates are vulnerable to bias [7, 8].

For UGI leaks specifically, effectiveness has not been synthesized across key clinical contexts, and implementation may depend on operator experience and access to specialized resources. Prior studies show limited standardization with respect to the index operation, oncologic versus nononcologic indications, leak location, and definitions of success, introducing methodological heterogeneity that complicates comparability and inference [9, [10].

This systematic review and meta-analysis evaluated the effectiveness and safety of EVT for anastomotic leaks after UGI surgery in adults.

## Materials and methods

### Study design and reporting

This systematic review and meta-analysis was reported in concordance with PRISMA 2020 [11].

### Data sources and search strategy

A comprehensive search of PubMed, Embase, Cochrane, and LILACS identified studies published from January 2015 through January 2025 that evaluated endoluminal negative-pressure therapy—hereafter endoscopic vacuum therapy (EVT)—for anastomotic leaks after upper gastrointestinal (UGI) surgery. Controlled vocabulary and free-text terms combined concepts for “anastomotic leak,” “upper gastrointestinal procedures,” and “endoluminal negative-pressure therapy.” Search strategies were tailored to each database; full strategies are presented in Appendix 1. Duplicates were removed in Covidence (Melbourne, VIC, Australia) [12]. Two reviewers (JD, SA) independently screened titles and abstracts and selected records for full-text review; disagreements were resolved by consensus or third-party adjudication. The last search was performed on June 20, 2025.

### Eligibility criteria

Observational studies published between January 2015 and January 2025 in English or Spanish were eligible if they reported clinical outcomes in adults treated with EVT (Endo-SPONGE or equivalent systems) for anastomotic leaks after UGI surgery, including esophagectomy, total or partial gastrectomy, segmental duodenal resections, sleeve gastrectomy, and Roux-en-Y gastric bypass. Randomized controlled trials were not included because no randomized trials assessing endoscopic vacuum therapy for upper gastrointestinal anastomotic leaks were identified during the systematic review process. Consequently, the evidence base was limited to observational studies reporting single-arm outcomes, which were treated analytically as case series. Exclusion criteria were ongoing studies, languages other than English or Spanish, single case reports, narrative reviews, letters, expert opinions, experimental studies, and interventions other than EVT. To ensure a homogeneous assessment of EVT efficacy, studies were included only if EVT was employed as the primary or first-line endoscopic treatment for the anastomotic leak. Studies reporting on leaks managed with combined endoscopic modalities (e.g., EVT concurrent with stent placement) or where EVT was used as a salvage therapy following failure of another primary intervention (e.g., suturing, clipping) were excluded. Data on the need for sequential additional endoscopic procedures following initial EVT (e.g., stenting, clipping) were extracted and reported as a secondary outcome.

### Data extraction

One reviewer (JD) extracted study characteristics using a standardized template (Appendix 2): first author, publication year, design (prospective or retrospective), sample size, surgical technique, surgical indication (oncologic or bariatric), leak location (intrathoracic or intra-abdominal), underlying pathology (benign or malignant), and EVT device (Endo-SPONGE or equivalent).

### Outcomes

The primary effectiveness outcome was successful leak closure, typically defined as endoscopic evidence of healed mucosa without residual cavity or fistula. Safety outcomes included overall complications, need for surgical re-operation, and mortality. Late complications included stricture and development of new fistula or abscess. Additional variables included length of stay, treatment duration, and number of sponge exchanges.

### Data synthesis and statistical analysis

When ≥ 2 studies reported comparable outcomes, meta-analyses were performed in R (R Studio version 2025.05.1 + 513; Posit Software, PBC). Proportions were pooled with random-effects models using the Freeman–Tukey double-arcsine transformation. As sensitivity analyses, logit transformation models were fitted to evaluate robustness. When feasible, subgroup analyses were conducted by index operation (e.g., esophagectomy, gastrectomy, Roux-en-Y gastric bypass), leak location, and risk-of-bias category. Studies that reported “upper gastrointestinal surgery” without procedure detail were classified as UGI. Studies with multiple procedures but without procedure-specific closure outcomes were grouped as mixed (MIX). When procedure-specific results were reported, strata were extracted separately to avoid unit-of-analysis errors. To assess the robustness of the pooled estimates and the influence of outliers, a pre-specified sensitivity analysis was planned by sequentially excluding studies with extreme effect sizes that were potential outliers. Publication bias was assessed visually with funnel plots and, where appropriate, with tests for small-study effects (e.g., Egger test) conditional on adequate study counts. Statistical heterogeneity was quantified with I2 and interpreted as low (< 25%), moderate (25–50%), or high (> 50%). Random-effects models were prespecified for substantial heterogeneity.

### Risk of bias assessment

Risk of bias for the included case series was assessed using the Joanna Briggs Institute (JBI) Critical Appraisal Checklist for Case Series [13]. This 10-item tool evaluates key methodological domains, including clarity of inclusion criteria, measurement of the condition, completeness and consecutiveness of participant inclusion, reporting of clinical information, and clarity of outcomes. Two reviewers independently appraised each study. Discrepancies were resolved by consensus. Studies were categorized based on the proportion of criteria met: low risk of bias (> 70% ‘Yes’ responses), moderate risk (50–70%), or high risk (< 50%).

### Certainty of evidence

Certainty of evidence was appraised using GRADE, considering study design, risk of bias, consistency, precision, and applicability, and rated as high, moderate, low, or very low.

The review protocol was prospectively registered in PROSPERO under the title “Endoscopic Negative-Pressure Therapy for Anastomotic Leaks After Upper Gastrointestinal Surgery: Systematic Review and Meta-analysis” (CRD420251208510).

## Results

### Study selection and characteristics

**Fig. 1:**
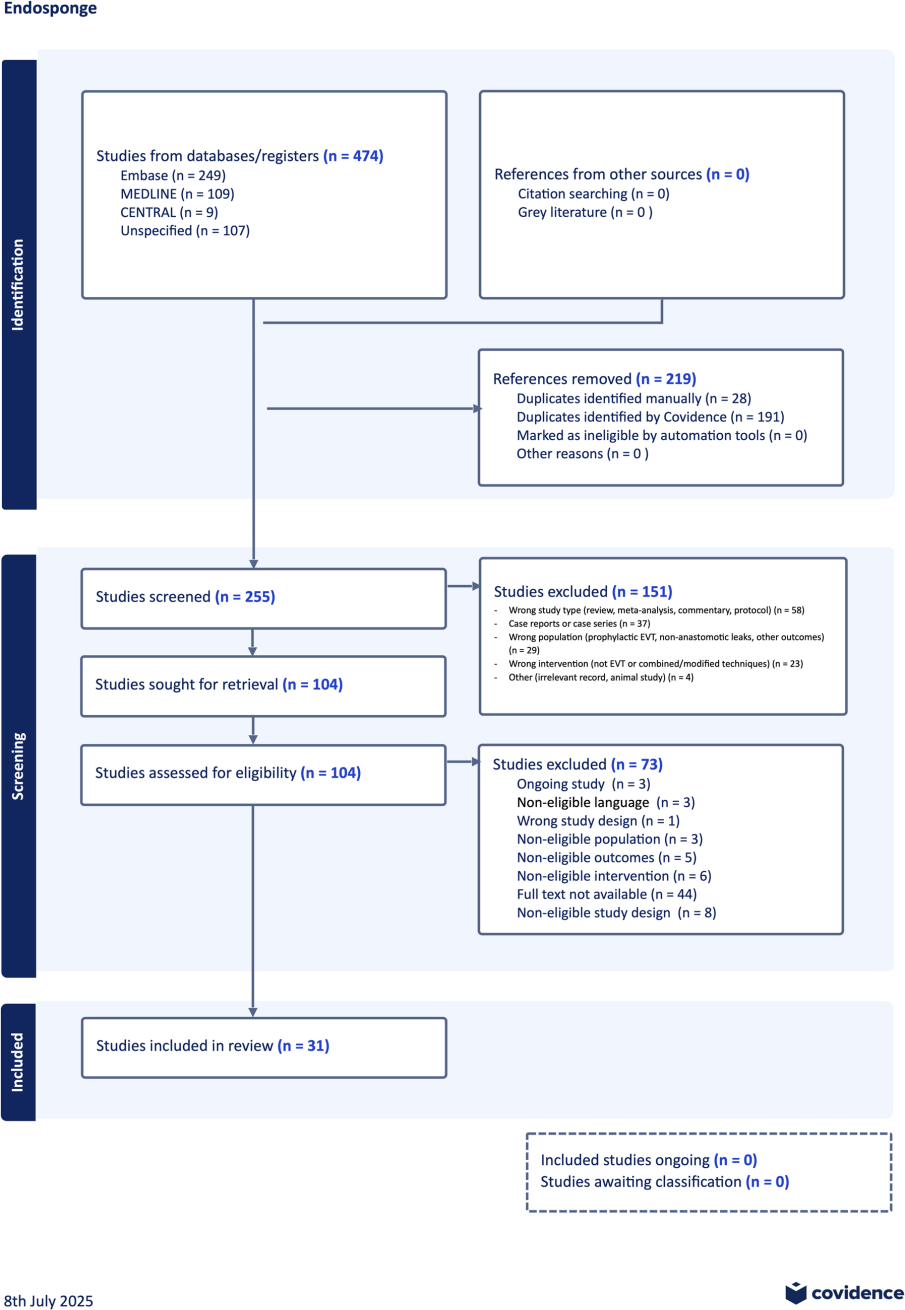
PRISMA 2020 fl ow diagram of the study selection process

The search yielded 474 records (Embase, 249; MEDLINE/PubMed, 109; LILACS, 107; Cochrane, 9). After deduplication, screening, and full-text assessment, 31 observational studies met eligibility criteria (Fig. 1). Most studies were retrospective (83.9%); five were prospective or mixed. Sample sizes ranged from 6 to 88 (median, 20), for a total of *n* = 767. Surgical contexts included esophagectomy, gastrectomy, bariatric procedures, and a smaller subset of unstratified UGI procedures. Eso-Sponge was the most frequently reported device; equivalent systems were described as EVAC or EVT. The predominant leak location was intra-abdominal. Detailed study characteristics are provided in Table 1 [14–44].

**Table 1:** Characteristics and clinical outcomes of studies on Endoscopic Vacuum Therapy (EVT) for anastomotic leaks after upper gastrointestinal surgery

Author (year)	Study design	Sample size (*n*)	Surgical technique	Type of surgery	Leak location	Device used	Overall success rate (*n*, %)	Complications (*n*)	Mortality (*n*, %)
Kuehn (2015)	Prospective	21	UGI	MIX	MIX	Eso-Sponge®	19 (90,5)	Mediastinitis 9 Empyema 3 Multiorgan failure 1 Stenosis 1	–
Mennigen (2015)	Retrospective	15	Esophagectomy	Oncologic	NR	Eso-Sponge®	14 (93,3)	0	1 (6,7)
Richter (2016)	Prospective	69	UGI	MIX	MIX	Eso-Sponge®	63 (91,3)	New abscess 3, Fistula 6, Bleeding 4, Peritonitis 1, Pneumonia 9, Mediastinitis 9, Pleural empyema 5, Sepsis 7, Renal failure 5, ARDS 3, Mediastinal emphysema 4, stenosis 8	4 (5,8)
Hwang (2016)	Retrospective	7	Ivor-Lewis 5, Total Gastrectomy 1, Segmental Esophageal Resection 1	Oncologic	Abdominal	EVAC	7 (100)	0	NR
Leeds (2016)	Retrospective + Prospective	9	Sleeve Gastrectomy	Bariatric	Abdominal	Eso-Sponge®	9 (100)	1 persistent pancreatitis, 1 uncontrol luminal contents	1 (11)
Smallwood (2016)	Retrospective	6	UGI	MIX	Intrathoracic (4), Abdominal (2)	Eso-Sponge®	6 (100)	0	0 (0)
Laukoetter (2017)	Prospective	54	UGI	MIX	MIX	EVAC	49 (94,2)	11 sponge dislocations and 5 minor bleedings	5 (52,9)
Noh (2018)	Retrospective	12	Ivor Lewis 7, Robotic Ivor Lewis 4, Others 1	Oncologic	MIX	EVAC	8 (66,7)	1 Anastomotic site bleeding	0 (0)
Berlth (2019)	Retrospective	27	Esophagectomy 20, Distal Esophageal Gastrectomy 3, Total Gastrectomy 4	Oncologic	MIX	EVAC	23 (85)	Short-term complications - 4/27 dislocation Long term complications - 1/27 stenosis	–
Min (2019)	Retrospective	20	Esophagectomy	Oncologic	Cervical (7), Intrathoracic (13)	EVAC	19 (95)	–	1 (5)
Archid (2020)	Prospective	8	Sleeve Gastrectomy	Bariatric	Abdominal	Eso-Sponge®	7 (87,5)	1 bleeding	–
Choi (2020)	Retrospective	11	Gastrectomy	Oncologic	Abdominal	CuraVAC®; CGBio Inc, Seongnam,	11 (100)	–	0 (0)
Jeon (2020)	Retrospective	22	Ivor Lewis 14, McKeown 4, Transthoracic Esophagectomy 2, Gastrectomy 2	Oncologic	Intrathoracic	EVAC	19 (86,4)	–	–
Sendino (2020)	Retrospective	11	Esophagectomy 7, Gastrectomy 2, Endoscopic Zenker’s Septotomy 1, Esophageal Repair after Boerhaave Syndrome 1	MIX	MIX	EVAC	7 (63)	1 Pleural Emphyema 1 retrofaringeal pain 3 stenosis	2 (17)
Archid (2021)	Retrospective	14	Sleeve Gastrectomy	Bariatric	Abdominal	Eso-Sponge®	12 (85,7)	2	1 (7,1)
DePasqual (2021)	Retrospective	7	UGI	MIX	MIX	EVAC	5 (62,5)	1 Bleeding	0 (0)
Hayami (2021)	Retrospective	23	Esophagectomy	Oncologic	–	EVT	19 (82,6)	–	0 (0)
JungCFM (2021)	Retrospective	23	Ivor Lewis 15, Gastrectomy 7, Gastrectomy + Distal Esophagectomy 1	Oncologic	Intrathoracic	EVT	18 (78,3)	–	1 (4,3)
Zhang (2021)	Retrospective	49	Transthoracic Esophagectomy	Oncologic	Intrathoracic	EVAC	42 (85,7)	- minor bleeding 1 - oxygen desaturation 1 - aspiration 1	4 (7,2)
JungCFM (2022)	Retrospective	27	UGI	Oncologic	MIX	EVT	23 (85,2)	–	–
El-Sourani (2022)	Retrospective	13	Ivor Lewis Esophagectomy	Oncologic	Intrathoracic	EVAC	12 (92,3)	–	5 (38)
Markus (2022)	Retrospective	20	Gastrectomy	MIX	Abdominal	Endo-Sponge System	18 (90)	–	–
Mastoridis (2022)	Prospective	7	UGI	MIX	MIX	EVT	6 (86)	–	–
El-Sourani (2023)	Retrospective	17	Esophagectomy	Oncologic	Intrathoracic	EVAC	14 (82)	Bronchial fistula 2 intestinal perforation 1	5 (29)
Lee (2023)	Retrospective	21	Esophagectomy	Oncologic	Cervical (7), Intrathoracic (14)	EVAC	19 (90,5)	–	0 (0)
Maier (2023)	Prospective	17	Esophagectomy	Oncologic	Cervical (8), Intrathoracic (9)	EVT	17 (71)	–	–
PattynamaLMD (2023)	Retrospective + Prospective	38	UGI	Oncologic	Cervical (5), Intrathoracic (27), Abdominal (6)	EVAC	28 (74)	2	3 (8)
Ascari (2024)	Retrospective	88	Ivor Lewis Esophagectomy	Oncologic	Intrathoracic	EVAC	39 (38,5)	–	6 (6,8%)***
Fahrenkrog (2024)	Retrospective	29	Ivor Lewis Esophagectomy	Oncologic	Intrathoracic	EVAC	24 (82,8)	–	2 (6.9)
Kollmann (2024)	Retrospective	42	UGI	MIX	MIX	EVT	42 (100)	–	–
Seo (2024)	Retrospective	40	Esophagectomy	Oncologic	Intrathoracic	EVT	33 (82,5)	–	–

^*^Average duration of endoscopic vacuum therapy: 28 days (range 5–48); 5 days in the case of Boerhaave syndrome and 26 days after endoscopic Zenker’s septotomy

^**^Average hospital stay: 45 days (range 10–90); 45 (26–90) days in cases of anastomotic dehiscence, 10 days in Boerhaave syndrome, and 54 days after perforation secondary to Zenker’s diverticulum septotomy

^***^Mortality expressed at 30 and 90 days after initiation of endoscopic vacuum therapy. Thirty-day mortality was 6 of 88 patients (6.8%) and 90-day mortality was 14 of 88 (15.9%)

### Primary outcome: effectiveness

**Fig. 2:**
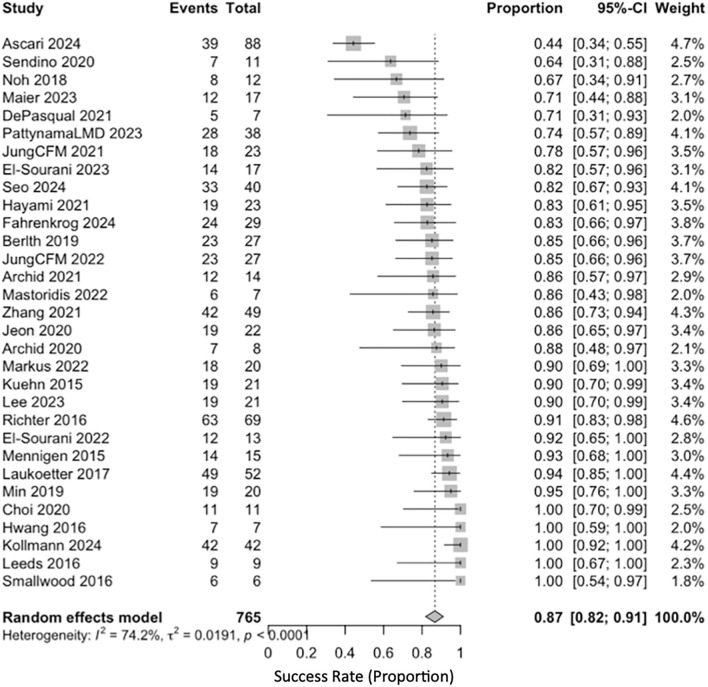
Forest plot showing pooled clinical success of EVT using a random-eff ects model (Freeman–Tukey transformation)

**Fig. 3:**
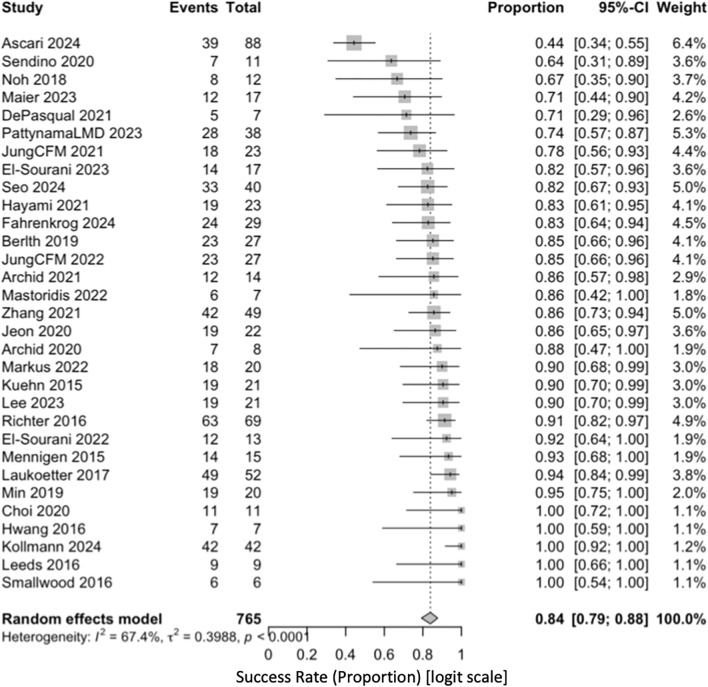
Sensitivity analysis using a logit transformation model for pooled clinical success

**Fig. 4:**
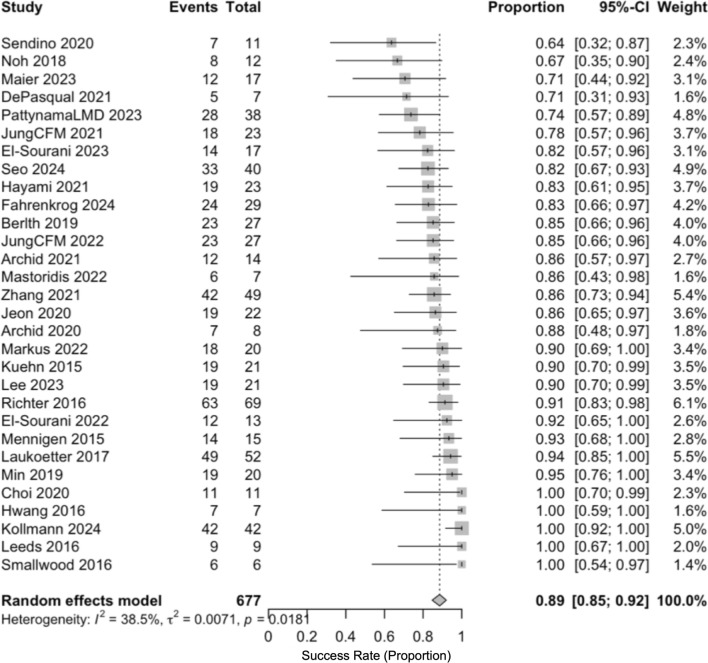
Sensitivity analysis excluding the outlier study, showing pooled clinical success and reduced heterogeneity

The pooled clinical success was 0.87 (95% CI, 0.82–0.91; *I*^2^ = 74.2%) (Fig. 2). In sensitivity analyses using a logit transformation, the estimate was 0.84 (95% CI, 0.79–0.88; *I*^2^ = 67%) (Fig. 3). One study (Ascaris 2024) was an outlier with a success of 0.44 (95% CI, 0.34–0.55). In a pre-specified sensitivity analysis to evaluate the influence of an outlier, this study was excluded. After its exclusion, the pooled success estimate remained high at 0.89 (95% CI, 0.85–0.97) with a reduction in heterogeneity (*I*^2^ = 38.5%), supporting the robustness of the primary finding (Fig. 4).

### Subgroup meta-analyses

**Fig. 5:**
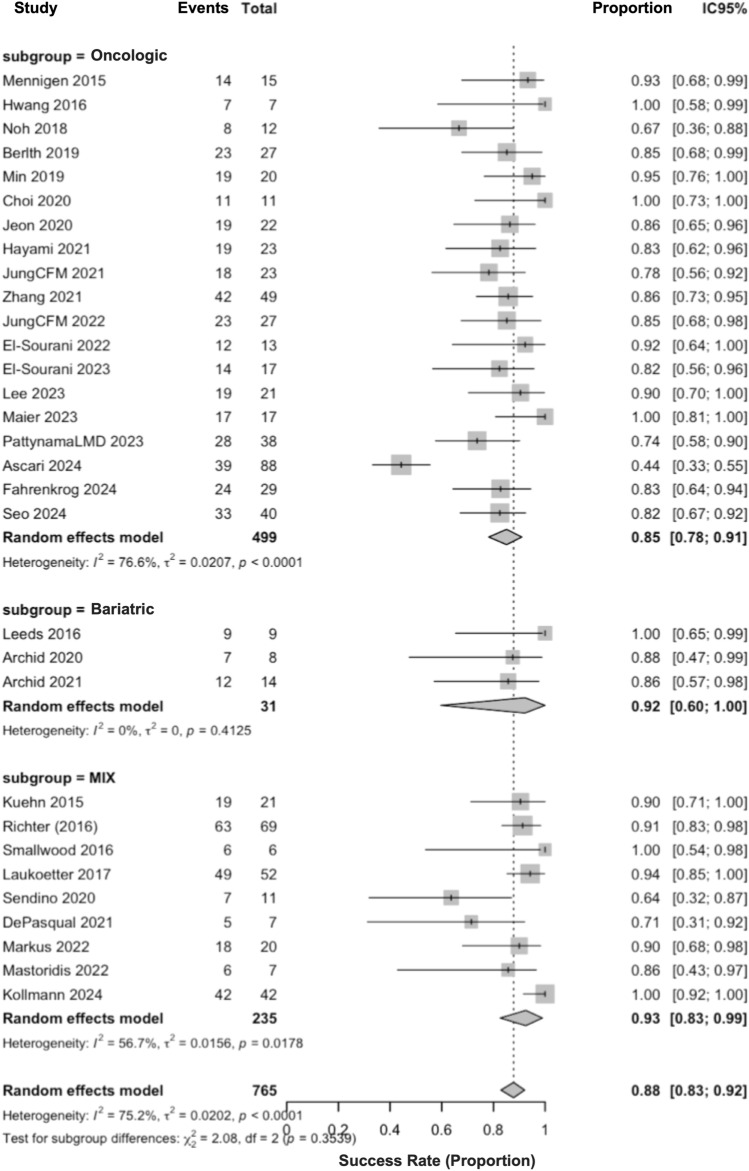
Subgroup analysis by surgical indication (oncologic, bariatric, mixed)

By surgical indication, pooled success was 0.85 (95% CI, 0.78–0.91) for oncologic series, 0.92 (95% CI, 0.60–1.00) for bariatric series, and 0.93 (95% CI, 0.83–0.99) for mixed, unstratified series (MIX). The test for subgroup differences was not significant (*P* = 0.35) (Fig. 5).

**Fig. 6:**
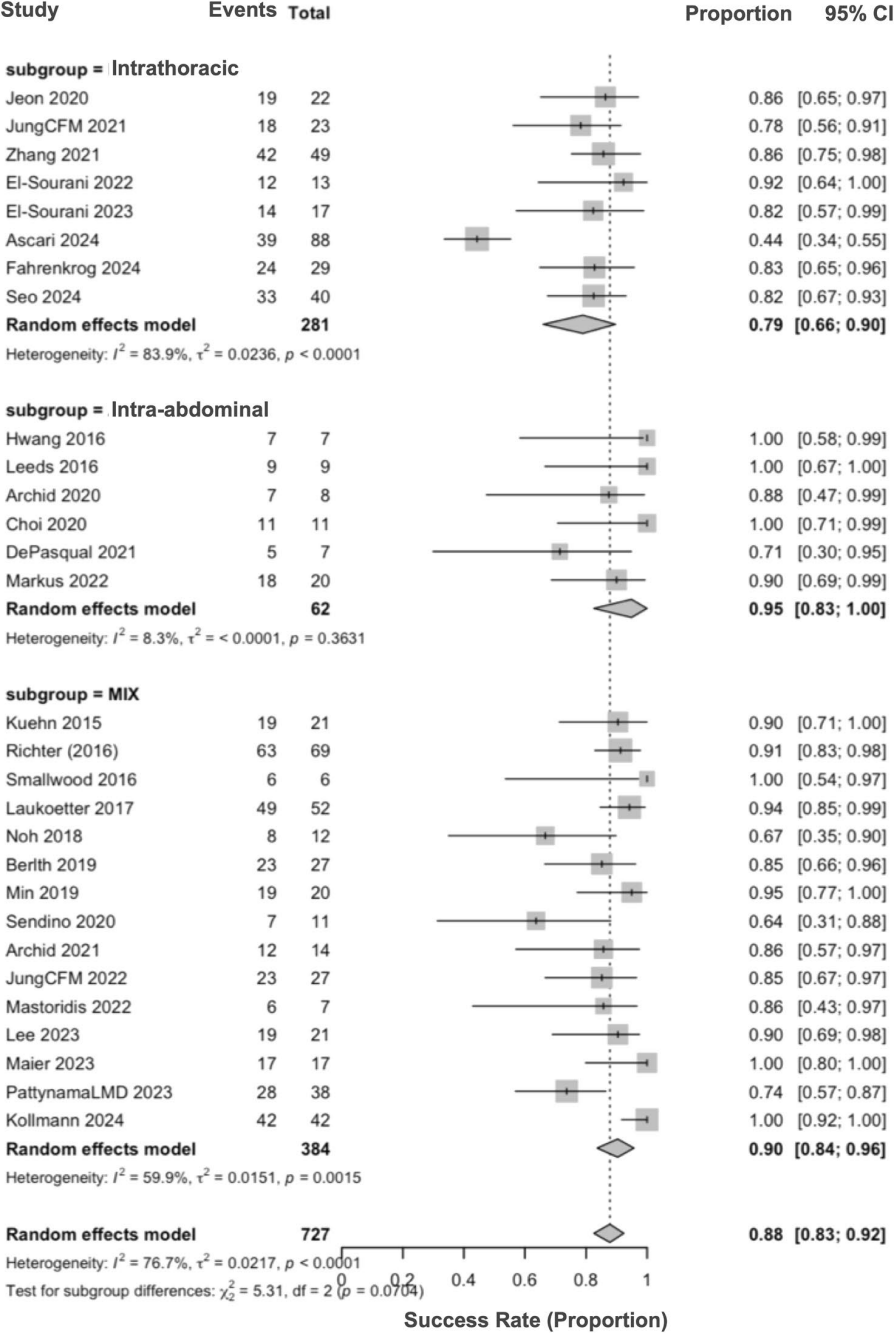
Subgroup analysis by leak location (intrathoracic, intra-abdominal, mixed)

By anatomic location, success was 0.79 (95% CI, 0.66–0.90; *I*^2^ = 83.9%) for intrathoracic leaks, 0.95 (95% CI, 0.83–1.00; *I*^2^ = 8.3%) for intra-abdominal leaks, and 0.90 (95% CI, 0.84–0.96; *I*^2^ = 59.9%) for mixed or unspecified locations; the between-subgroup test was not significant (*P* = 0.07) (Fig. 6).

### Secondary outcomes: safety

Safety reporting was heterogeneous and synthesized qualitatively. A total of 121 complications were documented. A detailed categorization of these events is presented in Table 2. Intrathoracic events included mediastinitis, empyema, and bronchial fistula; intra-abdominal events included intestinal perforation. Severe adverse events such as multiorgan failure were also reported. Late complications included anastomotic stricture in 34 of 364 (9%) and chronic fistula in 6 of 364 (1,6%). Surgical reintervention was reported in two studies, occurring in 7.1% (1/14) and 26.1% (18/62). Thirty-day mortality, available in 23 studies, was 7% (40/566). The procedural burden and resource utilization associated with EVT were heterogeneously reported across studies. Key parameters are summarized in Table 3. The number of sponge insertions (including initial placement) was reported in 20 studies (64%), with a typical median of 5 procedures per patient, though the range across all studies was wide (1–32). The total duration of EVT therapy, reported in 18 studies (58,1%), also varied substantially, with a median of approximately 23,5 days and a range from 1 to 142 days. Correspondingly, the median hospital stays, available from 21 studies (67,7%), ranged from 1 to 146 days (median approximately 24 days). The interval between sponge changes was specified in only 2 studies (6%), most commonly every 2 to 7 days. Data on additional endoscopic therapies (reported in 11 studies, 35,5%) or surgical revision (reported in 3 studies, 9%) were also inconsistent, with rates varying widely from 2 to 94% and 11,6 to 43%, respectively.

**Table 2:** Categorization and frequency of reported adverse events in patients undergoing Endoscopic Vacuum Therapy (EVT) for upper gastrointestinal anastomotic leaks

Category	Type of complication	*n* (% of 364)
Procedure-related	Sponge dislocation/migration	11 (3,02)
	Minor/local bleeding	13 (3,57)
	Oxygen desaturation/Aspiration	2 (0,54)
Systemic/infectious	Mediastinitis/Empyema	27 (7,41)
	Pneumonia/Sepsis	16 (4,39)
	Organ failure (Renal/MOF)	6 (1,64)
	Fistulization (Bronchial/Intestinal)	8 (2,16)
Major/long-term	Major bleeding (Gastric vessel)	1 (0,27)
	Anastomotic stricture	34 (9)
	Other (e.g., pancreatitis, uncontrol luminal contents)	3 (0,82)
Overall	–	121 (33)

**Table 3:** Summary of reported parameters on procedural burden and resource utilization

Parameter	Studies reporting *n* = 31 (%)	Summary of reported values	Notes
Sponge insertions per patient	20 (64%)	Reported median: 5 Overall range: 1–32	Includes initial placement. Values derived from reported medians, means, or ranges
Changing interval (days)	2 (6%)	Reported median: 5,75 days Reported range: 2–7 days	Few studies specified this parameter consistently
Duration of therapy (days)	18 (58,1%)	Reported median: 23,5 days Overall range: 1–142 days	From first EVT placement to confirmed closure
Median hospital stay (days)	21 (67,7%)	Reported median: 24 days Overall range: 1–146 days	Total hospitalization including the EVT period
Surgical revision	3 (9%)	Reported median: 10 Range of rates: 11,6%–43%	Extracted only from studies with clear numerator/denominator. Few studies specified this parameter consistently
Additional endoscopic therapies	11 (35,5%)	Reported median: 5 Range of rates: 2%–94%	Highly variable, depending on study protocol and leak complexity

*EVT* endoscopic vacuum therapy

The “Reported median” represents a qualitative synthesis of the most frequently reported central tendency across studies. The “Overall range” reflects the minimum and maximum values reported in any study

These estimates should be interpreted cautiously given reporting heterogeneity and differences in case mix.

### Risk of bias and certainty of evidence

**Fig. 7:**
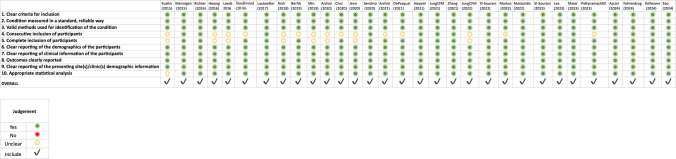
Risk of bias assessment using the Joanna Briggs Institute (JBI) checklist for case series

**Fig. 8:**
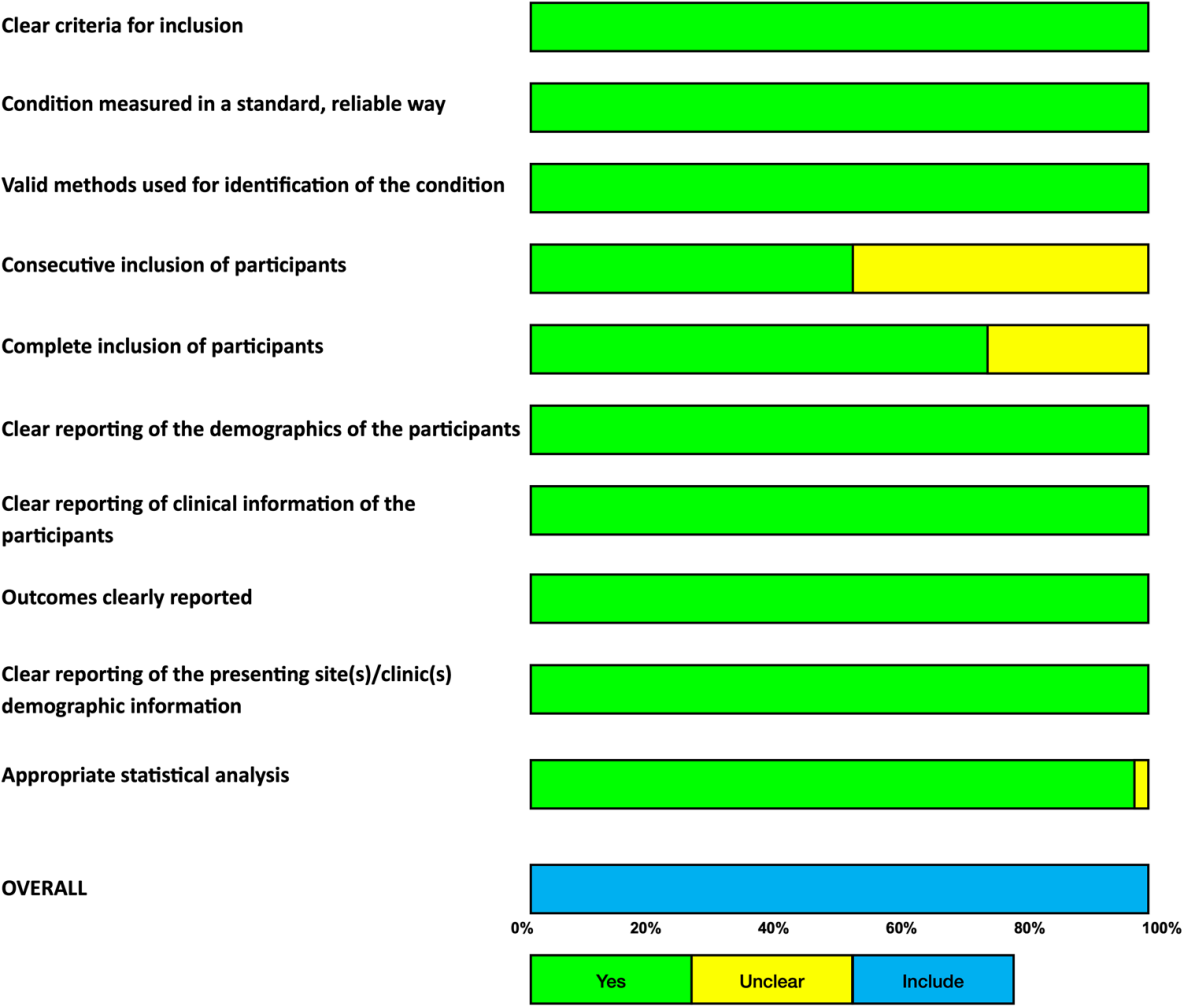
Summary of JBI appraisal showing the proportion of studies meeting each methodological criterion

The appraisal using the JBI checklist revealed a generally high standard of clinical reporting, though key methodological domains showed variability. While statistical methods were robust across the cohort (97%), the primary source of bias related to participant recruitment. Only 52% of studies (*n* = 16) explicitly confirmed the consecutive inclusion of participants, and 75% verified the completeness of inclusion. For the remaining studies, these domains were rated as ‘unclear’, reflecting a common limitation in retrospective and multicenter registries where reporting granularity is often insufficient to confirm rigorous enrollment protocols. Based on our pre-specified thresholds, 30 studies were classified as low risk, and one study was moderate risk of bias. A summary of the JBI appraisal is presented in Fig. 7**.** A comprehensive summary of the JBI appraisal, showing the percentage of studies meeting each criterion, is presented in Fig. 8. Certainty of evidence for clinical success was rated low by GRADE (Table 4), downgraded for risk of bias, inconsistency, and suspected small-study effects.

**Table 4:** GRADE of certainty of evidence

Outcome (measured as)	No. of studies (*n*)	No. of studies (*n*)	Risk of bias	Inconsistency	Indirectness	Imprecision	Publication bias	Relative effect (95% CI)	Absolute effect (95% CI)	Certainty
Clinical success (complete closure; proportion)	31 (767)	Observational	Moderate*	Serious (I2 ≈ 74%)	Not serious	Not serious	Suspected	–	870 per 1000 (820 to 910)	Low ⊕ ⊝ ⊝

*a. JBI APPRAISAL: Downgraded one level. While 97% of studies employed robust statistical methods and 30/31 were rated as low overall risk of bias, a specific limitation was identified in participant recruitment: only 52% explicitly confirmed consecutive inclusion, introducing a potential risk of selection bias

**Fig. 9:**
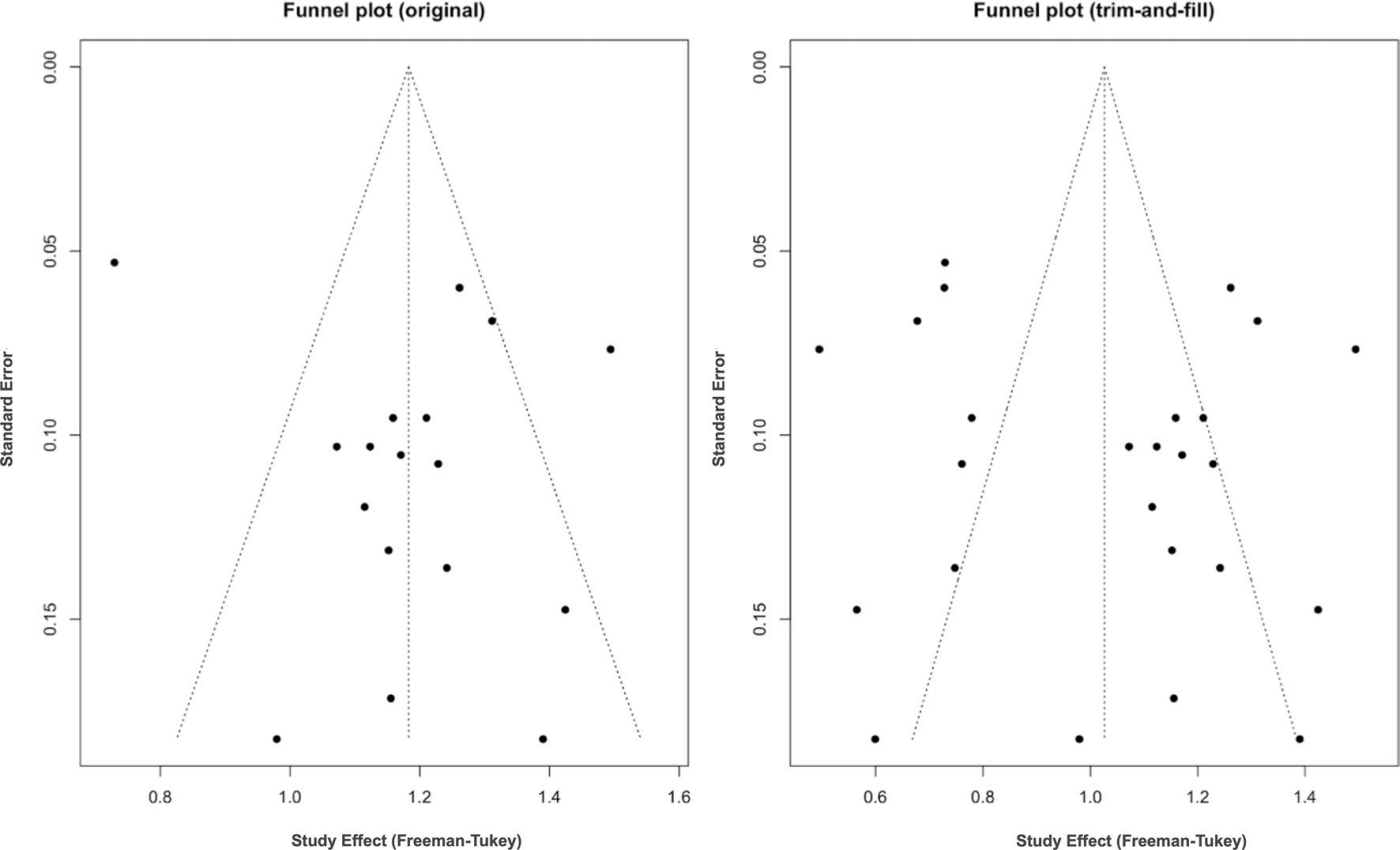
Funnel plot assessing publication bias and small-study effects

Random-effects pooled success was 0.87 (95% CI, 0.82–0.91), concordant with the logit-based sensitivity estimate of 0.84 (95% CI, 0.79–0.88). Inconsistency was downgraded due to high heterogeneity (I2 ≈ 74%). Indirectness was not downgraded. Imprecision was not downgraded given relatively narrow pooled CIs. Publication bias was suspected: the funnel plot showed mild asymmetry, and trim-and-fill slightly reduced the pooled estimate from 87 to 86% (Fig. 9).

## Discussion

This meta-analysis found a pooled clinical success of 87% (95% CI, 82–91%) for closure of anastomotic leaks after upper gastrointestinal (UGI) surgery with endoscopic vacuum therapy (EVT). Sensitivity analyses using a logit transformation and exclusion of an outlier yielded concordant estimates (84 and 89%, respectively), supporting robustness of the primary finding. These results align with prior reviews that reported success rates ≥ 80% [45].

Effectiveness appeared consistent across surgical contexts. For example, Tavares et al. reported EVT closure in 79.5% after esophagectomy and 90% after gastrectomy [46], and Scognamiglio et al. observed higher healing with EVT versus self-expandable metal stents (SEMS) (OR, 2.47) and a lower stricture rate (OR, 0.22), with no differences in mortality or length of stay [47]. In the present analysis, oncologic, bariatric, and mixed series yielded similar success (85, 92, and 93%, respectively), and differences were not statistically significant. Although intrathoracic leaks showed lower success than intra-abdominal leaks (79 vs 95%), the between-group comparison did not reach significance (*P* = 0.07), consistent with the greater technical demands of managing mediastinal collections.

One included study (Ascaris 2024) reported an atypically low success (38.5%), likely reflecting a complex cohort dominated by type II–III leaks after Ivor Lewis esophagectomy and prolonged hospitalization. Excluding this study reduced heterogeneity without materially changing the pooled estimate, further supporting stability of the overall effect.

Complications were variably reported but generally uncommon. Major adverse events included severe hemorrhage and rare aorto-esophageal fistulae; minor events included sponge migration, minor bleeding, and anastomotic stricture. In a 59-patient series, the most frequent events were sepsis (37.3%), pleural empyema (8.3%), mediastinitis (3.4%), tracheobronchial or lymphatic fistulas (5.1%), and stricture (3.4%); one patient (1.7%) experienced major bleeding from an aorto-esophageal fistula [46, 48]. Across studies with adequate reporting, anastomotic stricture occurred in 12% (34/294) and chronic fistula in 9% (6/63). Thirty-day mortality was 7% (40/566) across 23 studies. While clinically relevant, these data do not suggest a disadvantageous safety profile relative to more invasive alternatives.

Device-specific differences were not evident. Commercial systems (e.g., Eso-Sponge, Endo-SPONGE) and assembled setups were associated with comparable outcomes, suggesting that negative pressure is the principal therapeutic mechanism rather than any proprietary configuration. Formal cost-effectiveness analyses were rare; however, given high success and low reintervention rates, cost-effectiveness is plausible in centers with requisite expertise. In one report, per-patient costs were estimated at USD 10,188 in the operating room and USD 4528 in the endoscopy suite, with 3–5 sponge exchanges contributing to expenditure [46]. Furthermore, any comprehensive economic assessment must account for the substantial costs associated with the extended hospitalization required for EVT management. Our analysis found a reported median hospital stay of 24 days (range, 1–146 days). In the context of managing severe postoperative leaks, the daily hospitalization cost is a major driver of total expenditure. For instance, in the management of complex esophageal anastomotic leaks, the daily cost has been estimated to range from approximately $1000–$2000 USD for a surgical ward to $2000–$4000 USD for intensive care [49]. Applying a conservative estimate of $2000 USD per day to the median hospital stay of 24 days yields an approximate hospitalization cost of $48,000 USD per patient. This figure significantly surpasses the reported procedural costs of EVT itself (e.g., $4500–$10,000 USD), underscoring that the total economic burden of anastomotic leak management is dominated by the duration of inpatient care rather than the endoscopic intervention. This highlights an urgent need for future cost-effectiveness analyses that integrate both direct procedural costs and the indirect costs of prolonged hospitalization. This highlights an urgent need for future cost-effectiveness analyses that integrate both direct procedural costs and the indirect costs of prolonged hospitalization.

This review has limitations inherent to its evidence base. Although the JBI appraisal found a low risk of bias in most included case series (30/31), the certainty of evidence is rated low due to the observational nature of all studies and the substantial statistical heterogeneity observed. Small samples (median, 20) and inconsistent reporting of defect size, time to EVT initiation, and treatment duration limited granularity. Heterogeneity was high and the funnel plot suggested small-study effects; trim-and-fill reduced the pooled estimate minimally (from 87 to 86%) without altering interpretation. Our eligibility criteria require EVT to be the primary endoscopic intervention. While this ensures the pooled success rate reflects the efficacy of EVT as a first-line strategy and avoids confounding from combined therapies, it also means our findings cannot be generalized to its use as a salvage therapy after other modalities have failed. The high success rate observed (87%) should therefore be interpreted within this specific clinical context. Despite these constraints, convergence of subgroup results, absence of device-specific differences, and consistent sensitivity analyses support EVT as an effective therapeutic option for UGI anastomotic leaks, particularly in centers with established endoscopic-surgical collaboration. Prospective cohorts and randomized trials are warranted to refine indications, identify predictors of response, and evaluate cost-effectiveness.

## Conclusions

Across observational studies, endoscopic vacuum therapy (EVT; Endo-SPONGE or equivalent systems) achieved high clinical success for closure of anastomotic leaks after upper gastrointestinal surgery, with low rates of major complications. The pooled estimate was 87% and remained consistent in subgroup and sensitivity analyses. However, the evidence base is predominantly observational and heterogeneous, with risk of bias and low certainty. EVT is a reasonable option in centers with multidisciplinary endoscopic and surgical expertise. Key uncertainties include predictors of response and cost-effectiveness. Prospective cohorts and randomized trials are warranted to refine indications, standardize outcomes and definitions, and optimize implementation.

## Supplementary Information

Below is the link to the electronic supplementary material.

Supplementary file1 (DOCX 12 kb)

## Data Availability

The data underlying this meta-analysis—including the study-level extraction sheet, and analysis code—are available in [RSMA-EVAC] at [https://l1nk.dev/wDvDb], under a [license]. PRISMA 2020 flow diagram and checklist are provided as Supplemental Digital Content.
