# Supplementary material for: Endoscopic negative-pressure therapy for anastomotic leaks after upper gastrointestinal surgery: systematic review and meta-analysis
Source: Surg Endosc. 2026 Apr 20;40(5):3666–82. doi: 10.1007/s00464-026-12790-w (PMC13161319; doi:10.1007/s00464-026-12790-w)
Supplement: Supplementary file 1 — Supplementary file1 (DOCX 12 kb) [file 464_2026_12790_MOESM1_ESM.docx]

**Supplemental Digital Content (SDC)**

The PRISMA-2020 checklist and flow diagram are provided as Supplemental Digital Content at [<https://l1nk.dev/wDvDb>]
